# Investigating the impact of an online hydration intervention in care homes using the COM-B model: a mixed methods study

**DOI:** 10.1186/s12877-023-04515-8

**Published:** 2023-12-08

**Authors:** Danielle McMichael, Patricia Gillen, Caroline McGeary, Muhammad Sartaj, Lynsey Patterson

**Affiliations:** 1 Public Health Agency, 12–22 Linenhall Street, Belfast, BT2 8BS Northern Ireland; 2 Ulster University, Shore Rd, Newtownabbey, BT37 0QB Northern Ireland; 3 Health and Social Care Trust, Rosedale, 10 Moyallen Road, Gilford, BT63 5JX Northern Ireland; 4 Centre for Public Health, Institute of Clinical Sciences, Royal Victoria Hospital, Queen’s University Belfast, Belfast, BT12 6BA Northern Ireland

**Keywords:** Care home, Staff, Hydration, Fluid intake, Intervention, Multi-component, COM-B model, Antimicrobial Resistance

## Abstract

**Background:**

Antimicrobial resistance is a serious threat to public health. To reduce antimicrobial resistance, interventions to reduce gram-negative infections, specifically urinary tract infections, are vital. Early evidence suggests increased fluid intake is linked with a reduction in UTIs and subsequently has potential to reduce antibiotic usage. Care homes have a high prevalence of UTIs and provide an opportunity in a closed setting to deliver an intervention focused on increasing fluid intake, where it is supported and monitored by health care workers. The study aimed to evaluate the impact and feasibility of an online staff focused intervention over a 30 day period to increase the hydration of care home residents with a view to reducing the burden of AMR in this setting.

**Methods:**

The study was a pre and post intervention with a sequential explanatory mixed methods design. The intervention was delivered online in 3 care homes, containing 3 main components underpinned by the COM-B model including hydration training, 7 structured drinks rounds and a hydration champion to change behaviour of care home staff. A pre and post questionnaire assessed the impact of the intervention on staff and data was collected on fluid intake, drinks rounds delivered to residents, UTIs, antibiotic used to treat UTIs, falls and hospitalisation. Descriptive statistics summarised and assessed the impact of the study. Focus groups with care home staff provided qualitative data which was thematically analysed.

**Results:**

Staff increased in self-perceived knowledge across the six components of hydration care. 59% of residents had an increase in median fluid intake post intervention. During the time of the intervention, a 13% decrease in UTIs and antibiotic usage to treat UTIs across the 3 care homes was recorded, however falls and hospitalisations increased. Themes arising from focus groups included the role of information for action, accessibility of online training, online training content.

**Conclusions:**

This study demonstrates that a brief, low cost, online multi-component intervention focused on care home staff can increase the fluid intake of residents. A reduction in UTIs and antibiotic consumption was observed overall. Empowering care home staff could be a way of reducing the burden of infection in this setting.

## Background

Antimicrobial Resistance (AMR) is a worldwide public health issue [1]. It is estimated that by 2050 AMR will cause 10 million deaths each year, a reduction in 2-3.5% in gross domestic product, and cost up to 100 trillion dollars, across the world [1]. In response, strategic action plans have been published by the World Health Organisation (WHO) [2] and reflected locally [3, 4]. An independent review of AMR highlighted that tackling gram-negative bacterial (GNB) infections, which drive a significant amount of antibiotic consumption, will be key to reducing AMR [1]. In response, the United Kingdom (UK) government have set ambitions to halve healthcare associated GNB bacteria blood stream infections; delivering a 25% reduction by 2021–2022 and 50% reduction by 2023–2024; the Northern Ireland (N.I) government signed up to this ambition [3].

One of the most common infections caused by GNB are urinary tract infections (UTIs); in 2019 it was estimated there were 405 million (95% CI 359–447) cases worldwide and subsequently 237,000 related deaths [5]. The prevalence of UTIs increase with age [6–8]. It is also known that UTIs cause a significant burden in care homes [7, 9–11]. The higher burden of infection in care homes and subsequent antibiotic consumption, have led some to suggest that they could serve as a reservoir for resistant infections [8, 12].

A number of factors have been associated with an increased risk of UTIs including current health status, residential status, previous or current catheterisation, previous antibiotic use, and most recently poor fluid intake [8, 13]. Fluid intake is a potentially modifiable risk factor and has therefore been chosen as a target for intervention, to reduce UTIs and subsequently antimicrobial consumption. In the care home setting, healthcare workers have a key role providing support for residents with daily tasks such as eating, drinking and washing. They therefore present an ideal target group to influence the fluid intake of residents. Older people are at a higher risk of being dehydrated due factors such as a reduced sense of thirst, immobility, lower urine concentrating ability, and conditions such as dementia, which may lead to forgetting to consume adequate fluids [14]. While increasing fluid intake can be a potential way to reduce the risk of developing a UTI, particularly in this population, there are a range of challenges specifically with data collection methods in such settings. For example, maintaining records of fluid intake or how fluid intake is measured. When modifying a risk factor which is dependant upon personal preference such as types of drinks or requirements for conditions such as dysphasia and dementia it is important to understand these issues can affect the impact of an intervention or the findings of such an intervention [15].

To achieve this, healthcare workers need to be empowered with knowledge around the benefits of fluid intake and afforded the opportunity to support residents to monitor and improve their fluid consumption. The success of interventions to modify behaviour can be optimised by using the COM-B model, which is at the core of the Behavioural Change Wheel [16]. The model breaks down the components of behaviour: capacity, opportunity, and motivation, and puts these drivers at the forefront of the development of the intervention [17].

The aim of this project was to evaluate the impact and feasibility of an online staff focused intervention over a 30 day period to increase the hydration of care home residents with a view to reducing the burden of AMR in this setting.

## Methods/Design

### Study design

The study was a pre and post intervention with a sequential explanatory mixed methods design, which included a pre and post staff questionnaire and an online education intervention. The hydration intervention was based on the I-Hydrate tool kit, a validated tool kit for delivering information to staff on improving hydration practices in care homes [18]. Approval was sought from the author to use and adapt the study materials, including the online videos and questionnaire [18]. Adaptations to the questionnaire were piloted for acceptability with two healthcare staff.

The methodology of this study was influenced by the impact of the COVID-19 pandemic. The online delivery of the intervention was key to ensure it could still be delivered due to changes in the operation of care homes, including the limitation of visitors accessing care homes. A number of considerations were made when finalising the methodology such as the amount of data being collected, the ease of collection, the time frame of pre and post intervention periods, and the amount of time required to complete training. The needs of the study were balanced with the need avoid potential negative or unintended consequences that could arise from increasing the workload of staff in the context of managing a vulnerable population in a closed setting during a pandemic.

### Care home / staff recruitment

Convenience sampling was used to identify three care homes to participate in the study. All three were managed by the same company and provided different levels of service for residents; one provided a mix of both nursing and residential care (care home one; CH1), the second solely provided care specifically for people with dementia (CH2) and the third provided a mix of both care for people with nursing (CH3 general nursing; CH3.G.N) and dementia care needs (CH3 dementia unit; CH3.D.U). Care home staff were self-referred by their management team. Inclusion criteria included registered and unregistered staff working in the care homes, who normally assist residents with their fluid intake (n = 142). Staff who did not directly provide fluid intake support for residents were excluded.

### Intervention

#### Preliminary meetings – care home management and care home staff

Preliminary meetings were held on Zoom with senior care home managers. A presentation provided an overview of the study followed by a discussion to: (1) ensure that the intervention was feasible; (2) agree the data to be collected, and; (3) ask for volunteers to be hydration champions [18]. In addition, a gate keeper letter was provided with the requirements of the study, participant information sheets (PIS) and consent forms for staff. Further to this, three introductory meetings were held with care home staff on Zoom to explain the study, how to access training and how to use the data collection tools.

#### Baseline measures (pre-intervention)

To understand the opportunities to support fluid intake, a review of the care home groups current nutritional care policies and practices was undertaken to establish normal practices within the care home.

A previously validated 9-item pre -intervention self- administered questionnaire was used to establish the self-perceived knowledge and understanding of staff in relation to hydration before the intervention [18]. The anonymous questionnaire was administered on Qualtrics and a link disseminated to care home staff by a manager.

An aggregated summary of the age and gender balance of care home residents was collected to understand the care home demographics. Anonymised baseline data of specific resident outcomes was established using records held by the care homes including fluid intake of residents, number of falls, number of urinary tract infections, number of prescriptions for antibiotics to treat UTIs and number of hospital admissions.

### Implementation of intervention

#### Phase 1: online hydration training

The online hydration training, aligned to the capacity component of the COM-B model, aimed to improve the self-perceived knowledge of staff with the use of five short online videos from the I-Hydrate tool kit [18]. The videos ranged in length from approximately 3–8 min, and included introductions to hydration care, offering choices for fluid intake, protecting drinking times, supporting individuals with dysphagia and supporting individuals in seating and positioning for drinking fluids.

#### Phase 2: 7 structured drinks rounds & Hydration Champion

Phase 2 was implemented over a 4-week period in each care home. Seven structured drinks rounds were carried out at seven intervals each day using drinks, and foods that are high in fluid. The drinks rounds created a physical opportunity for staff to utilise their knowledge to support residents to improve their hydration based on their individual needs, addressing the opportunity component of the COM-B model [19, 20]. To record the delivery of the seven structured drinks, a data collection sheet was developed based on the data collection tool used in a previous study [19]. Training on use of the tool was provided to care home staff.

Finally, focusing on the motivation component of the COM-B model, at least one hydration champion per care home was identified to motivate and empower staff. The hydration champion was a designated member of staff in each care home to provide motivation and support to staff to carry out training, deliver the 7 structured drinks rounds, and a link to feedback to management should they require support from the research team.

#### Phase 3 post-intervention assessment

The key metrics collected at baseline were reassessed and further questions to evaluate the intervention were added, again utilising previously validated tools [18]. The anonymous questionnaire was re-administered on Qualtrics, and the link was disseminated to care home staff by a manager.

Focus groups were held with staff from the care homes to gain further insight into their experience with the impact of the multi-component intervention. The questions and prompts were structured using the literature, responses to the survey and the components of the COM-B model. Sessions were held on Zoom and an experienced qualitative researcher was present at each focus group. Four focus groups lasted approximately 15–30 min; two were conducted for registered staff and two for unregistered staff and each session included 2–4 staff members. Staff volunteered to participate. The focus group sessions were recorded and transcribed verbatim by an external transcriber.

### Data analysis

Quantitative data from the pre and post training staff questionnaire and the data from the seven structured drinks rounds were analysed using descriptive statistics including numbers/percentages. To assess individual fluid intake and change over the pre and post periods the median was used to reduce the impact of missing data, as an equal number of days of fluid intake was not reported pre and post intervention. Fluid intake data was only presented for residents present pre and post intervention. Finally, the characteristics of the care home residents were described.

The thematic analysis of the focus group transcripts was guided by Braun and Clarke [21] and carried out separately by DM and PG to ensure independent analysis and increase rigour and trustworthiness [21, 22]. Responses were aligned to the respective component of the COM-B model.

Analysis was conducted for quantitative data using R (version 3.6.1) and Microsoft Excel 2010.

### Ethics

Ethical approval was obtained from Ulster University’s Institute of Nursing and Health Research Ethics Filter Committee (FCNUR), (Reference: FCNUR-20-030). The study was staff focused and informed consent was obtained before proceeding with participation or data collection.

## Results

### Care home resident demographics

Across the 3 care homes almost 70% of residents were female and over 90% of residents were older than 65 years old (Table 1). There were 142 care home staff across the 3 care homes: 34 registered staff and 108 unregistered staff.

**Table 1:** Care home resident demographics including residents age, sex and total residents by care home

	Total	n	%
CH1	**35**		
Sex			
Male		9	25.7
Female		26	74.3
Age			
Under 65		8	22.9
Over 65		27	77.1
CH2	**40**		
Sex			
Male		12	30.0
Female		28	70.0
Age			
Under 65		0	0.0
Over 65		40	100.0
CH3.G.N	**30**		
Sex			
Male		12	40.0
Female		18	60.0
Age			
Under 65		1	3.3
Over 65		29	96.7
CH3.D.U	**17**		
Sex			
Male		7	41.2
Female		10	58.8
Age			
Under 65		0	0.0
Over 65		17	100.0
All Care Homes	**122**		
Sex			
Male		40	32.8
Female		82	67.2
Age			
Under 65		9	7.4
Over 65		113	92.6

### Staff demographics and self-perceived knowledge

The response rate for the pre and post questionnaires was 67% (n = 94/142) and 60% (n = 86/142) respectively. Staff who responded to both questionnaires came from a range of job roles with Health Care Assistants being the most common (68.1% and 73.3%, 64/94 and 63/86 respectively). The working experience of staff ranged from less than 1 year to more than 10 years (Table 2).

**Table 2:** Pre and post intervention self-administered questionnaire results

	Pre Questionnaire			Post Questionnaire		
	Total	n	%	Total	n	%
Care home	**94**			**86**		
CH1		24	25.5%		31	36.0%
CH2		19	20.2%		17	19.8%
CH3 (CH3.G.U & CH3.D.U)		46	48.9%		34	39.5%
Unknown		5	5.3%		4	4.7%
Job role	**94**			**86**		
Domestic Staff		9	9.6%		5	5.8%
Health Care Assistant		64	68.1%		63	73.3%
Management		3	3.2%		2	2.3%
Nurse		15	16.0%		13	15.1%
Other		2	2.1%		2	2.3%
Unknown		1	1.1%		1	1.2%
Years worked in care home	**94**			**86**		
< 1 Year		21	22.3%		28	32.6%
> 10 Years		12	12.8%		11	12.8%
1–2 Years		23	24.5%		14	16.3%
3–5 Years		21	22.3%		16	18.6%
6–10 Years		17	18.1%		17	19.8%
Previous fluid intake training	**94**			**86**		
No		36	38.3%		34	39.5%
Yes		57	60.6%		52	60.5%
Unknown		1	1.1%		0	0.0%
Confident in supporting residents with their individual fluid intake needs	**94**			**86**		
1- Not Very Confident		2	2.1%		1	1.2%
2		1	1.1%		1	1.2%
3		4	4.3%		1	1.2%
4		26	27.7%		21	24.4%
5- Very Confident		61	64.9%		61	70.9%
Time to spend with residents to support them with their individual fluid intake needs	**94**			**86**		
I feel rushed most of the time		16	17.0%		12	14.0%
I have time to give residents all the support they need		21	22.3%		19	22.1%
I have time to give residents all the support they need/ most of the time but rushed on occasion		1	1.1%		0	0.0%
Most of the time but rushed on occasion		35	37.2%		32	37.2%
Sometimes but I feel I need more time		20	21.3%		21	24.4%
Unknown		1	1.1%		0	0.0%

Almost 40% of staff who responded in the pre (38.3%, n = 36) and post questionnaire (39.5%, n = 34) reported that they had not previously had training in providing fluid intake support to the residents (Table 2). Staff who responded to both questionnaires had a range of experience; 22.3% (n = 21) of staff had < 1 years’ experience and 12.8% (n = 12) of staff had > 10 year’ experience (Table 2). While experience of staff varied, almost 50% of staff in the pre and post questionnaires had less than 3 years’ experience. However, most felt confident (at the baseline assessment) when supporting individual residents with fluid intake with 64.9% (n = 61) of staff responding that they felt “very confident” increasing to 70.9% post – intervention (n = 61).

**Fig. 1:**
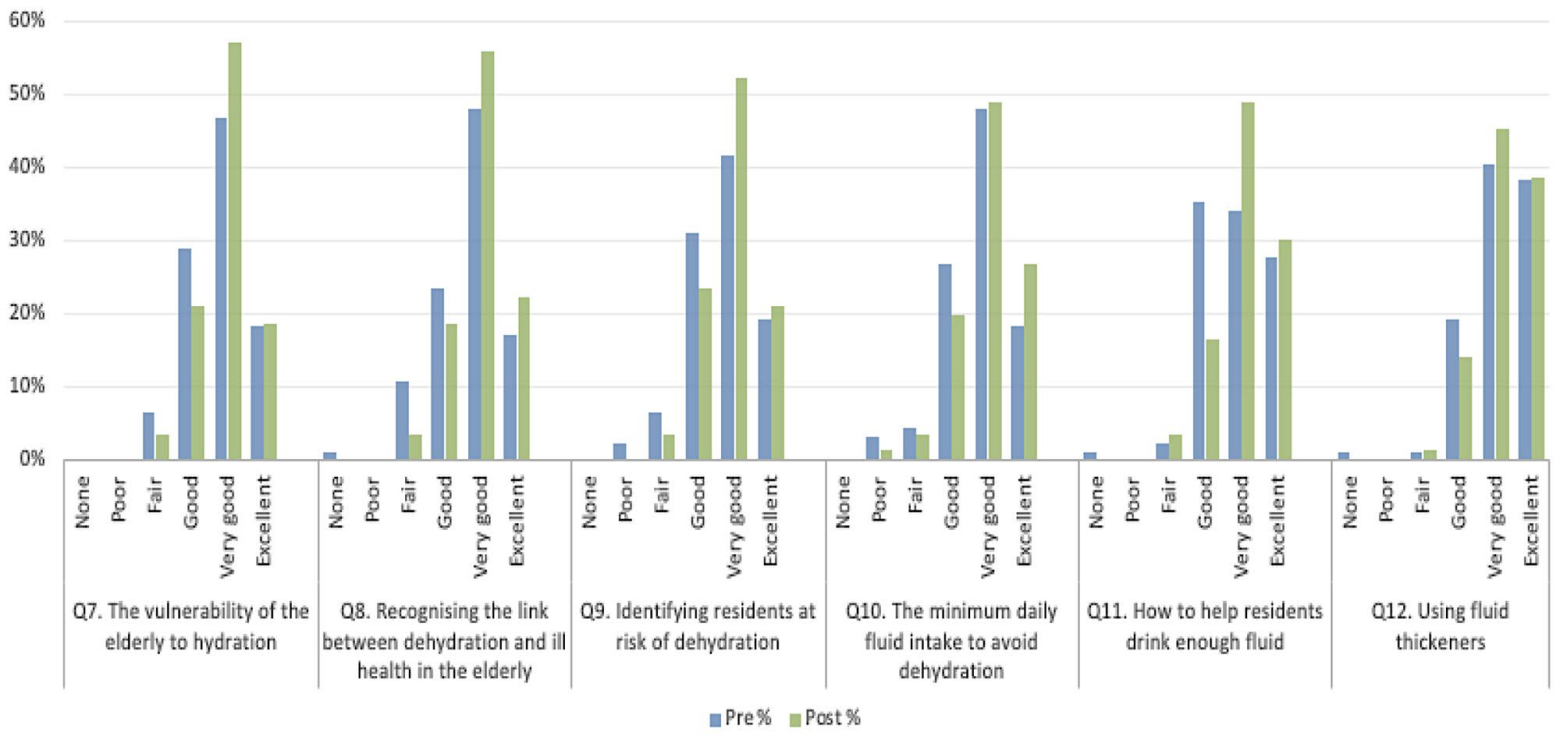
Pre and post intervention self- reported self-perceived knowledge of the six components of fluid intake support

Figure 1 demonstrates the increase in staff reporting their self-perceived knowledge following online hydration training as “Very good” or “Excellent” across all six components of hydration care. The median score across the six components of hydration was “Very good” pre and post intervention indicating that staff self-assessed their self-perceived knowledge reasonably high pre-intervention.

### Fluid intake

Across the 3 care homes 59% (n = 70/118) of residents had an increase in median fluid intake post intervention (Table 3). However, in CH2, 57.5% (n = 23/39) of residents had a decrease in median fluid intake. The increase in resident’s median fluid intake post intervention across the 3 care homes ranged from 12.5 ml to 875 ml. Where the median fluid intake for residents decreased post intervention this ranged from − 10 ml to – 425 ml. In residents who did not previously meet their individual fluid intake target, 48.6% (n = 17/35) met their target post intervention. In CH3.G.N, of the 11 who did not meet their target pre or post intervention, 9 residents had an increase in their median fluid intake ranging from 25ml to 190ml (Table 3).

**Table 3:** Care home resident’s maximum and minimum median fluid intake and achievement against fluid intake target

	Pre intervention			Post intervention		
	Total	N	%	Total	N	%
CH1						
Median Resident Fluid Intake- Minimum (ml)		1162.5			1105	
Median Resident Fluid Intake- Maximum (ml)		1995			2520	
No. Residents Reaching Individual Fluid Target	**35**	30	85.7	**35**	33	94.3
CH2						
Minimum Median Resident Fluid Intake (ml)		850			745	
Maximum Median Resident Fluid Intake (ml)		2070			2060	
No. Residents Reaching Individual Fluid Target	**39**	33	84.6	**39**	30	76.9
CH3.G.U						
Minimum Median Resident Fluid Intake (ml)		975			1025	
Maximum Median Resident Fluid Intake (ml)		1500			1700	
No. Residents Reaching Individual Fluid Target	**27**	8	29.6	**27**	16	59.3
CH3.D.U						
Minimum Median Resident Fluid Intake (ml)		850			932.5	
Maximum Median Resident Fluid Intake (ml)		1750			1700	
Residents Achieving Individual Fluid Intake Target	**17**	12	70.6	**17**	14	82.4
All Care Homes						
Minimum Median Resident Fluid Intake (ml)		850			745	
Maximum Median Resident Fluid Intake (ml)		2070			2520	
No. Residents Reaching Individual Fluid Target	**118**	83	70.3	**118**	93	78.8

The number of UTIs and antibiotics used to treat UTIs decreased by 13% across all three care homes (Table 4). CH1 had a 63% decrease in UTIs and antibiotics used to treat UTIs; the highest number of UTIs occurred in CH2, where most residents had a decrease in median fluid intake (Table 4). An antibiotic was prescribed for each UTI in the pre and post intervention periods (Table 4). Falls increased by 183% and hospital admissions increased by 200% post intervention period (Table 4). 36% (n = 5/14) of those with UTIs in the post intervention period had a decrease in the median fluid intake ranging from − 25 ml to -190 ml, where median fluid intake increased in residents who had a UTI in the post intervention period the range was 20ml to 300ml.

**Table 4:** Pre & Post Key Metrics- UTIs, Antibiotics for UTIs, Hospitalisations & Falls

	Pre Intervention (n)	Post Intervention (n)	+/- (n)	Percentage Change (%)
CH1				
Falls	1	3	2	200
Hospital Admissions	2	5	3	150
UTIs	8	3	-5	-63
Antibiotics prescribed for UTIs	8	3	-5	-63
CH2				
Falls	3	4	1	33
Hospital Admissions	1	1	0	0
UTIs	4	6	2	50
Antibiotics for UTIs	4	6	2	50
CH3.G.N				
Falls	0	4	4	NA
Hospital Admissions	0	5	5	NA
Utis	0	3	3	NA
Antibiotics for UTIs	0	3	3	NA
CH3.D.U				
Falls	2	6	4	200
Hospital Admissions	1	1	0	0
UTIs	4	2	-2	-50
Antibiotics for UTIs	4	2	-2	-50
All care homes				
Falls	6	17	11	183
Hospital Admissions	4	12	8	200
UTIs	16	14	-2	-13
Antibiotics for UTIs	16	14	-2	-13

### Evaluation of the online hydration training

**Fig. 2:**
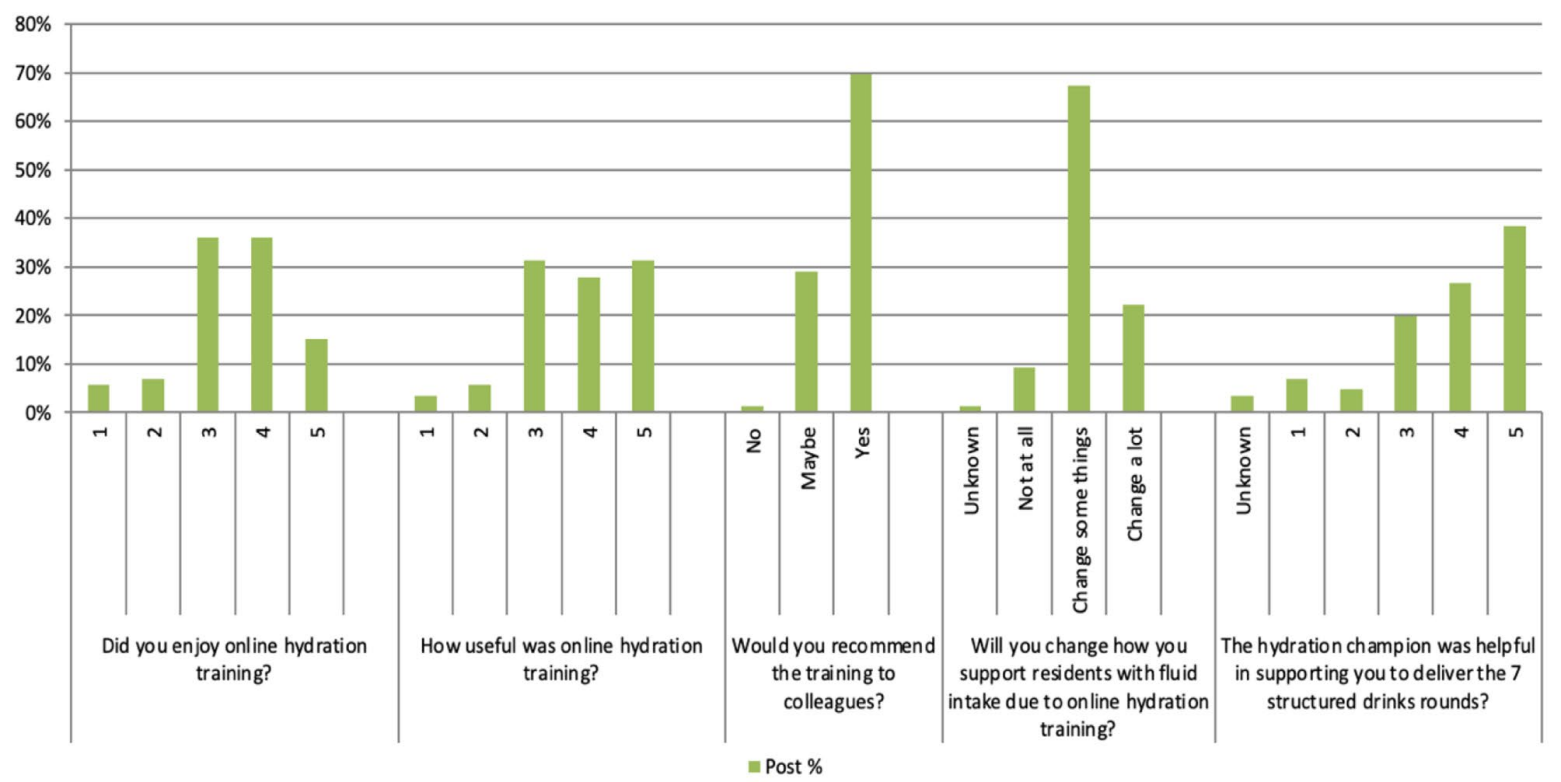
Post intervention questionnaire evaluation of the 3 main components of the intervention

The evaluation of the online hydration training indicated that 15.1% (n = 13/86) of respondents “enjoyed it a lot” and 36% (n = 31/86) “enjoyed it” (Fig. 2). When evaluating the usefulness of the online hydration training, 27.9% (24/86) found it “useful” and 31.4% (27/86) found it “very useful”. Most respondents also reported that they would recommend the online hydration training to colleagues (69.8%, n = 60/86). Following the online hydration training 22.1% (19/86) of staff would “change a lot” and 67.4% (58/86) would “change some things” in their daily practice when supporting residents with fluid intake (Fig. 2).

### Focus groups

#### The accessibility of online training

Staff reported that the online training was easily accessible, and it was useful particularly for the working patterns and pressures experienced by care home staff. However, some staff still prefer or enjoy elements of face-to-face training such as group interaction and demonstrations.

*“It was OK online, but sometimes I think that face to face is easier. Maybe you can have better discussions. Because when you’re not very good on this kind of technology… but other than that it was fine. I didn’t have a problem with it.”*. *FG1-Nurse*

Staff also found it was useful that online training was accessible at their convenience and that it could be accessed retrospectively should they want to look over certain aspects of the training.

*“Yeah, I felt the same. Being able to access it when you wanted to. For different people who are working different times, maybe night duty sleeping during the day, if you missed the link initially, you can go in and just check. So I find online good.” FG2-Nurse*.

#### Online training content

Online hydration training content was informative and useful, and it improved the understanding and confidence of staff in supporting residents with fluid intake. Staff felt that the content covered the main aspects of hydration care and supported them to carry out the intervention.

*“Well I found them really, really useful because I’ve only been a carer for just two and a half years now… It just helps you understand everything a wee bit more, doesn’t it?… Giving you more confidence in being able to provide care for our wee residents, which is upmost and very important. But they were really helpful, so they were.” FG3-Health care assistant (HCA)*.

#### Barriers to accessing training

While there was consensus between staff that training was both accessible in an online format and the content encompassed the main concepts of how to support fluid intake, there are still barriers to accessing training. Some staff members reported that this isn’t their only job or if they were working felt they didn’t have as much time to access the training.

*“Yeah, it was in my case. I was usually working or it just wasn’t the right time. I have other commitments as well, outside of work.” FG3-HCA*.

#### The impact of seven structured drinks rounds

Seven structured drinks rounds more opportunities during the day for residents to drink and the individual support helped with residents who are still independent in drinking. The focus on the rounds and delivering support with intake also had a positive impact on the accuracy of recording.

*“Going and actually standing and staying with him, to have his drink, instead of leaving it with him. Even though he’s completely compos mentis and able to do for himself, but the fact that we were with him, it did help to get him drinking.” FG1-Nurse*.

#### Challenges of delivering seven structured drinks rounds

Staff found that the delivery of seven structured drinks rounds could be challenging with certain residents and despite their best efforts it may not always be successful if residents refuse to drink. While encouraging residents with fluid intake was important to staff it was also important that they respect the resident’s choice if they refuse a drink especially when persistent intervention can lead to irritation, particularly with residents who have dementia.

*“I generally work in a dementia unit. You just find with some of them, because of their dementia, they just didn’t like intervention sometimes. And it was more of an irritation to them. I’m thinking of one resident in Particular. If she won’t drink or eat, you just have to leave her be.”. FG3-HCA*.

The consensus on seven structured drinks rounds was that it was beneficial for staff and residents. Although completing different documentation for each resident can be challenging. Staff had ideas on how to reduce the burden and that despite having slightly more work to do, agreed that it is achievable.

*“to have an individual file on everybody… or chart for everyone in their own files… it would probably be done a lot easier then.” FG3 – HCA*.

#### The role of information for action

Staff found that the increase in documenting fluid information was beneficial, and they could discuss fluid intake of residents with hydration champions, this led to staff taking action to support and prioritised with low fluid intake. Information supported staff morale as they could identify if they had been able effectively support fluid intake.

“it’s more documented, I suppose that’s what I’m trying to say. And it’s interesting, really, to see, from our champions when they report back to us, how much they have actually taken. Which is good because there’s sometimes as a carer you sort of feel, have you done enough?” *FG3 – HCA*.

#### The impact of hydration champions on motivation

Hydration champions highlighted the challenge in keeping staff motivated, they were well received by staff and acted as role models to other staff.

*“No, that was OK. It just was a bit difficult making sure you got … the time goes in so quickly because there’s so many different things to attend to. It was… at times it was a bit of a challenge, depending on what staff you had on duty, keeping them motivated.” FG1-Nurse*.

Hydration champions maintained awareness and focus of other staff on the intervention and encouraged teamwork.

*“But I think it brought it to mind more that you should be doing it more often. Just to keep going round and topping them up every so often. Even people in their rooms, residents in their rooms, to make sure that they are getting their drinks as well.” FG1-Nurse*.

#### 7 Structured drinks rounds

Staff reported that the opportunity and time to support residents with their individual fluid intake requirements remained similar pre and post intervention. Most staff felt they had the opportunity to support residents “most of the time but rushed on occasion” (37.2% and 37.2%, 35/94 and 32/86 respectively). The proportion of staff who felt “rushed most of the time” decreased slightly post intervention (17% and 14%, 16/94 and 12/86 respectively) (Table 2).

Table 5 highlights that only 26% (n = 4,952/19,107) of the seven structured drinks rounds were recorded as delivered. The data for seven structured drinks rounds was linked to fluid intake data at a person level and no clear concordance was identified between the number of drinks rounds reported as delivered and the amount of fluid intake. The role of the hydration champions in supporting the delivery of 7 structured drinks rounds was found to be supportive for most staff (56%, n = 73/86).

**Table 5:** Seven structure drinks rounds delivery outcomes by care home

Care home	CH1		CH2		CH3 G.N		All care homes	
	n	%	n	%	n	%	n	%
No. residents recorded	35	38.5	36	39.6	20	22.0	91	100
†Total potential drinks rounds	7350	100	7560	100	4200	100	19,110	100
Drinks round delivered	779	10.6	2008	26.6	2158	51.4	4945	25.9
Drinks round not delivered	6298	85.7	3135	41.5	2008	47.8	11,441	59.9
Not recorded	30	0.4	2283	30.2	34	0.8	2347	12.3
§Resident in hospital	238	3.2	133	1.8	0	0	371	1.9
§Resident asleep	5	0.1	1	0	0	0	6	0

7 structured drinks rounds were collected for a 30-day period

Data unavailable for CH3.D.U

§ Yes/no were the only responses to be recorded in the data collection sheet – where additional information has been captured this has been recorded and presented

† Total potential drinks rounds that could have been delivered to residents - for each resident 210 drinks could have been offered during the intervention; this was multiplied by no. of residents in care home

## Discussion

The study found that, overall, hydration increased across the three care homes and the number of UTIs and antibiotics received decreased, following a short online training intervention. It demonstrates the potential for online hydration training to increase staff self-perceived knowledge of the six main components of hydration care. Staff supported the online delivery of training and although baseline self-perceived knowledge was rated as very good, this did increase post-intervention. Hydration champions were also generally well received and helped sustain staff motivation. This suggests that this multi-component intervention could present a low-cost method to address the training gap that was identified, with regards to hydration.

While the intervention was targeted at staff, the beneficiaries were intended to be the residents. The intervention increased fluid intake in 60% of residents with a number who previously did not meet their fluid intake. While overall intake increased in two of the three care homes, for the third care home there was a notable reduction in fluid intake. This care home, care home two, solely provided care for people with dementia and it is conceivable that even with structured drinks rounds, it is challenging for this specific group of residents if they refuse to drink. Indeed, staff reported that dementia patients often became irritated if they persisted with their attempts to support fluid intake, and it was important to staff that they respected the residents wishes. Nonetheless, staff recognised the benefits of more drinks being offered and highlighted that they used the information they had collected to identify residents who had low fluid intake, which is a benefit of using the seven structured drinks rounds [23]. While overall there was a decrease in UTIs across the three care homes, the numbers did fluctuate which reflects the short duration of the intervention and the small number of participants. The data collection around antibiotic use showed that for every UTI reported an antibiotic was prescribed. Reassuringly, the antibiotics prescribed were all included within the ‘Access’ group of WHO’s AWaRe categorisations, which work against many susceptible pathogens and have a lower potential for resistance [24].

One unexpected finding was the increase in falls and hospitalisations post intervention. This is difficult to interpret, but might reflect changes in care home practices in response to the COVID-19 pandemic and the increased pressures and reduction in staffing due to the impact of COVID-19 self-isolation periods [25]. Indeed, during this study visiting was reintroduced in care homes and the third wave had just commenced, which is likely to have impacted on staff workload and staffing pressures [25].

The identification of hydration champions worked well, with staff reporting that they were visible, discussed roles with staff and provided advice about the drinks rounds. They motivated other staff to get involved and it led to unregistered staff taking on more responsibility for supporting fluid intake of residents, helping to alleviate work pressures, and acknowledging that the structured drinks rounds can be time consuming. This is in line with previous findings in which role modelling and mentoring junior staff were key for sustainability [19, 26].

Another key theme emerging from the focus groups was that staff were motivated by feedback from the data they had collected. Staff noticed differences in fluid intake and the hydration champion also provided feedback, placing a conscious focus on hydration care. Despite challenges with the pressure of increased documentation, staff noted that it was possible to deliver this, and they felt it benefited residents and staff. Indeed, the role of quantifying improvement in motivating staff has been recognised [27]. Future research should prioritise how best to support data collection in care home settings and optimising the provision of feedback to staff.

The use of the COM-B model to guide the multi-component intervention highlights the various components that are required for behaviour change to occur. The components are low cost, support both staff and residents, easily adopted by care home staff and with little disruption to daily practice in most cases. The COVID-19 pandemic has changed working practices, and normalised the use of online video conferencing platforms in place of face to face meetings. For this study, we were able to fully engage with senior management in care homes and a range of care home staff to deliver the project successfully using video conferencing, training videos hosted online and online data collection platforms. This highlights the future opportunity to roll out projects to a larger number of participants, than may usually be feasible with a face-to-face approach, with a reduction in related costs. Barriers to online training can occur due to time pressures on staff and it is important that staff have time set aside during the working day to access online training, and not expected to be undertaken in their own time. Data collection can be challenging for staff, whose primary focus is the care and support of the residents. It was important that staff received appropriate training and that data collection tools are simplified with a clear purpose.

One of the strengths of this study was the use of the same questionnaire pre and post intervention. The same questionnaire was used in both this study and Green et al. [18], where it was used as an evaluation with retrospective pre assessment of self-perceived knowledge. This approach could reduce response shift bias observed in traditional pre and post testing and improve internal validity. However, it is advised to be used along with pre and post measurements rather than standalone [28–30]. Retrospective pre assessment of self-perceived knowledge also has the potential to decrease internal validity due to the impact of the implicit theory of change, in which participants believe pre-test scores should be lower than post-test [31]. Response bias can also occur where social desirability results in the participant feeling compelled to give the response they believe is expected [32]. These potential biases make a direct comparison for the outcomes of both studies difficult, however both face to face and online training appear to be effective in increasing hydration care self-perceived knowledge of care home staff. The limitations of this study are for the most part characterised by the difficulties in data collection in a care home setting. Challenges tend to be due to staffing pressure, the amount of data already collected and measuring fluid intake, which can lack reliability and objectivity [33–35]. It is accepted fluid intake data has lower validity in terms of actual intake but as the method remains consistent there would be an acceptable level of reliability to detect any change in intake. Alternative methods of measure such as observation may result in observer bias and can only account for limited time periods and small samples, while using drink diaries are not appropriate for residents who don’t have the capacity to complete them [35].

The care home group already had a clear and appropriate hydration policy in place with recommendations and a suggested structure for delivering fluids. This could have impacted the findings and make it more difficult to detect a change. The level of self-perceived knowledge in relation to hydration was already reasonably high at baseline with a median score of “very good”, although self-reported self-perceived knowledge may not be truly reflective of actual knowledge or lead to a change in behaviour.

Finally, this study was observational in nature and used a convenience sample and for that reason differences in fluid intake / outcome measures were not assessed statistically. Despite this, we did observe an increase in hydration and a reduction in UTIs, in keeping with a previous study [18]. This finding, in addition to the low cost of the intervention means there is potential for wider roll out of this intervention across care homes.

## Conclusions

During the time of the hydration intervention, a decrease in UTIs among care home residents was recorded, reducing subsequent antibiotic usage to treat UTIs. The study demonstrates that a brief multi-component intervention, targeted towards care home staff can be effective in increasing staff self-perceived knowledge of hydration care, and creates capacity, opportunity, and motivation for staff to support residents to increase fluid intake. The online nature of the project highlights the opportunity for the roll out of projects to a larger number of participants to increase impact and reduces the costs related with face-to-face projects.

## Data Availability

All data generated or analysed during this study is available on request.
